# Iterative Co‐Development of the Tripartite Joint Risk Assessment Operational Process and Tool Through Incorporating Information From Pilots in Indonesia, the United Republic of Tanzania, and Georgia

**DOI:** 10.1002/puh2.70398

**Published:** 2026-10-08

**Authors:** Elizabeth Mumford, Kaylee Errecaborde, Kachen Wongsathapornchai, Ryan Aguanno, Guillaume Belot, Dana Cole, Vasily Esenamanov, Dirk Pfeiffer, Ong‐orn Prasarnphanich, Xavier Roche, Ismaila Seck, Tieble Traore, Endang Wulandari, Sophie von Dobschuetz

**Affiliations:** ^1^ School of Veterinary Medicine University of Surrey Guildford UK; ^2^ World Health Organization Geneva Switzerland; ^3^ Food and Agriculture Organization of the United Nations Bangkok Thailand; ^4^ Food and Agriculture Organization of the United Nations Rome Italy; ^5^ United States Department of Agriculture Animal and Plant Health Inspection Service Fort Collins Colorado USA; ^6^ Health Emergencies Hub for the South Caucasus WHO Regional Office for Europe Tbilisi Georgia; ^7^ Centre for Applied One Health Research and Policy Advice City University of Hong Kong Kowloon Hong Kong SAR P. R. China; ^8^ Royal Veterinary College London UK; ^9^ World Health Organization, Regional Office for Africa—Dakar Emergency Hub Dakar Senegal; ^10^ World Health Organization Indonesia Country Office Jakarta Indonesia

**Keywords:** Joint Operational Tools, One Health, risk assessment, zoonoses

## Abstract

**Introduction:**

Zoonotic diseases pose risks to human, animal, and ecosystem health; effective management relies on coordinated actions across sectors. Although One Health principles promote collaboration across sectors, operational tools for multisectoral risk assessment (RA) are lacking. Therefore, the Tripartite (FAO, WHO, and WOAH) developed the first‐ever One Health operational tool for Joint Risk Assessment (JRA‐OT), with inputs from 15 pilot countries and international experts. This article describes how information from pilots in Indonesia, the United Republic of Tanzania, and Georgia contributed to informing co‐development of the final JRA process and tool.

**Methods:**

Three in‐person meetings with international RA and zoonotic disease experts with a One Health background were convened in Rome, Italy, to co‐develop the methodology (November 2017, September 2018) and incorporate pilot feedback (February 2020). Development criteria included a standard, systematic, qualitative, and multisectoral RA process, facilitating incorporation of expert opinion and sector‐specific RA results. The pilot workshops described here were conducted in Indonesia (March 2018), the United Republic of Tanzania (March 2019), and Georgia (July 2019), and feedback was incorporated iteratively into the JRA‐OT by a Tripartite Secretariat.

**Results:**

These pilot workshops demonstrated JRA's utility and provided critical information for improvements. Key findings included the (1) value of policy‐level risk framing, (2) importance of political will, (3) need for subnational engagement, (4) importance of including all relevant sectors, and (5) necessity of clear, stepwise guidance to facilitate efficient collaboration and support training of national trainers.

**Conclusions:**

Co‐development of the JRA using country pilots and expert consultations allowed for field‐testing and iterative improvement of the tool, ensuring incorporation of user requirements, robust methodology for the joint, multisectoral assessment of national zoonotic disease risks, and ownership by the different sectors. The process serves as a model for co‐development of other multisectoral operational tools.

## Graphical abstract

Piloting of the Tripartite Joint Risk Assessment (JRA) process and tool in countries provided feedback for improvement, evidence‐based estimates of national risks from priority zoonotic diseases, and practical training of national facilitators. The JRA offers simple step‐by‐step guidance to countries wishing to undertake joint qualitative risk assessments.

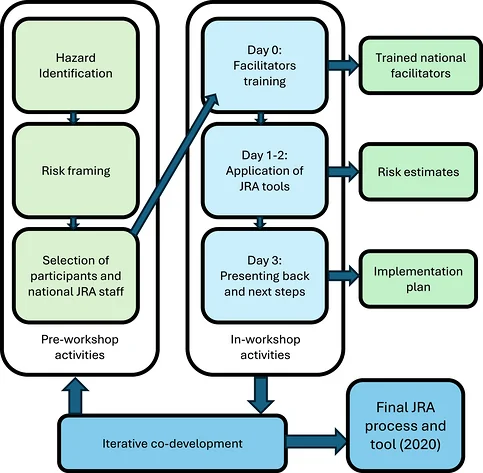

## 1 Introduction

Actions and policies to identify, assess, manage, and reduce risks from zoonotic diseases benefit from functional collaboration and engagement across stakeholders and sectors, including academia, the private sector, local communities, and government ministries responsible for human health, animal health, food safety, wildlife, environment, trade, education, security and disasters, and the economy [1]. Under the International Health Regulations (2005) monitoring and evaluation framework—Joint External Evaluation guidance [2], countries should have the capacity to conduct joint assessments of risk for zoonotic diseases using a One Health approach.

In 2014, countries concerned about the potential spread of a zoonotic avian influenza A(H7N9) virus strain in Asia wished to align the results of sectoral risk assessments (RAs) and risk‐related communication messages but encountered challenges in sharing information and conducting RA jointly across sectors. Due to its sensitive nature, information related to zoonotic diseases can potentially influence consumer behavior and/or compromise a country's economy or tourism if made public. Sharing such information between sectors often must be authorized and may be restricted or delayed, especially in the absence of clear sharing mechanisms or policies. Further, although countries could access sector‐specific RA methodologies and processes, they encountered challenges in assessing risks jointly when encountering differing steps and terminology across RA methods and the reluctance of sectors to agree on an approach unfamiliar to them in the absence of expert guidance. These countries requested assistance from the Tripartite [3] to identify and propose a technically sound approach to conducting joint, multisectoral zoonotic disease RA.

Technical aspects of estimating risks are, in principle, similar across RA methods, and it is normally possible to incorporate information from other sectors during sector‐specific zoonotic disease RAs. However, each sector‐specific method evolved to achieve different objectives. Consequently, RA methods are operationally different, do not easily align, and do not lend themselves to joint use. Although many institutions and agencies publish RA reports for zoonotic diseases, an extensive search of published guidance for conducting zoonotic disease RA across health‐related disciplines identified only sector‐specific tools available at that time. Further, the guidance from WHO [4], WOAH [5](updated in 2024) and FAO [6](available internally but unpublished at the time) referred to assessment for zoonotic diseases but had been developed exclusively by and for the human health and animal health sectors, respectively. The RA procedures of the *Codex Alimentarius* Commission [7] were also available but primarily focused on quantitative assessment of contaminants in food and were not practical for use by countries. To fill this gap, the Tripartite convened international RA experts and pilot countries to iteratively co‐develop the first‐ever One Health operational tool for conducting zoonotic disease RA jointly across sectors.

The Joint Risk Assessment (JRA) was published as the Joint Risk Assessment Operational Tool (JRA‐OT) in 2020 [8], as one of the operational tools supporting the Tripartite Zoonoses Guide [1]. The JRA‐OT provides a systematic and iterative step‐by‐step process to bring together stakeholders from different sectors. Together, they share expertise, discuss available information, and jointly identify and qualitatively assess risks associated with zoonotic hazards nationally and subnationally, based on strategic policy concerns. Benefits include the following:

- Facilitates sharing of information and expertise from all relevant sectors, increasing the technical validity of results, including evidence‐based management measures and communication messages.
- Facilitates agreement and ownership of results across sectors, allowing development of nuanced and acceptable management interventions with fewer negative externalities.
- Prospectively considers strategic policy needs across sectors at national and subnational levels, expediting aligned and contextualized implementation done jointly or separately by each sector.
- Fosters mutual understanding of perceptions, mandates, constraints, and daily work across sectors, improving communication for future collaborative activities, including emergency response.
- Identifies missing information, allowing targeting of surveillance, research, and capacity building.
- Facilitates political engagement and support for sustained One Health collaboration through establishment of a routine technical, output‐driven collaborative process located within a multisectoral coordination and governance infrastructure.

This article describes the iterative and collaborative process undertaken to co‐develop the JRA, which is an example of and a model for applied One Health, demonstrating:

- Initiation based on needs of the user community
- Balanced, shared leadership across multiple sectors
- Inclusion of expertise, perspectives, information, and experiences from across a broad, inclusive, and multi‐scalar scope of technical and policy stakeholders
- Iterative consideration and integration of knowledge insights acquired during implementation

The JRA itself exemplifies best practices in One Health implementation, namely,

- Developing a shared objective
- Using a standardized process to facilitate collaboration, reduce conflict, and achieve joint outputs
- Providing tangible outputs, specifically, consolidated hazard information, qualitative risk estimates, and options for risk‐based management and communication, that are immediately practical, relevant to local contexts, and useful for diverse stakeholders
- Building technical capacity
- Demonstrating transparency and inclusivity
- Promoting trust, awareness, and relationship‐building across sectors and scales

Here we describe national pilots in Indonesia, the United Republic of Tanzania, and Georgia, selected on the basis of their importance in informing the final JRA process and tool. Our findings serve as a model to inform co‐development of other multisectoral operational tools by applying an inclusive and consultative process consistent with the One Health approach.

## 2 Methods

### 2.1 Initial Tool Development

The JRA‐OT development began in 2014 and was led by a multisectoral international team of epidemiologists, zoonotic disease experts, and RA experts from six countries in three regions, including many from this team of authors, convened through virtual meetings. They reviewed and examined existing sector‐specific RA methods and tools for their suitability and relevance and agreed on the following criteria for the joint RA:

1. Provide a standard, systematic process for conducting evidence‐based RA on joint priority zoonotic disease hazards.
2. Be qualitative and facilitate incorporation of expert opinion when only minimal and/or poor‐quality data are available.
3. Allow identification of data gaps, and benefit from iterative use as additional data becomes available.
4. Promote use of data and results from sector‐specific RAs.
5. Provide step‐by‐step operational guidance to facilitate effective and equitable collaboration and sharing of information, expertise, and perspectives.
6. Be prospectively joint across all relevant sectors, that is, public health (e.g., ministries of health, public health agencies), animal health (e.g., veterinary services), environmental (e.g., wildlife health, environmental protection agencies), and others, such as security and disaster services.
7. Function within existing zoonotic disease management and One Health governance infrastructures.
8. Be adaptable to the relevant context
9. Be usable by governments, academia, or anyone wishing to conduct RA jointly across sectors.
10. Support international sector‐specific RA and health security frameworks (e.g., International Health Regulations [9], WHO Rapid RA [4], WOAH Import Risk Analysis [5], FAO draft Guidelines on Rapid RA [now published [6]]).

Once the new basic JRA process and tool were agreed, they were tested during (1) two international in‐person workshops in Rome, Italy (November 2017, September 2018), bringing together international multisectoral RA experts from different geographical regions selected by the three organizations to review and fine‐tune the methodology, and (2) 15 country pilots conducted between March 2018 and December 2019 (Figure 1), which guided iterative updates. All pilots were in‐person and had the same direct and indirect objectives (Table 1). A final in‐person expert consultation was held in Rome, Italy (February 2020), and the JRA‐OT was published in December 2020 [8]. The development timeline is depicted in Figure 2.

**FIGURE 1:**
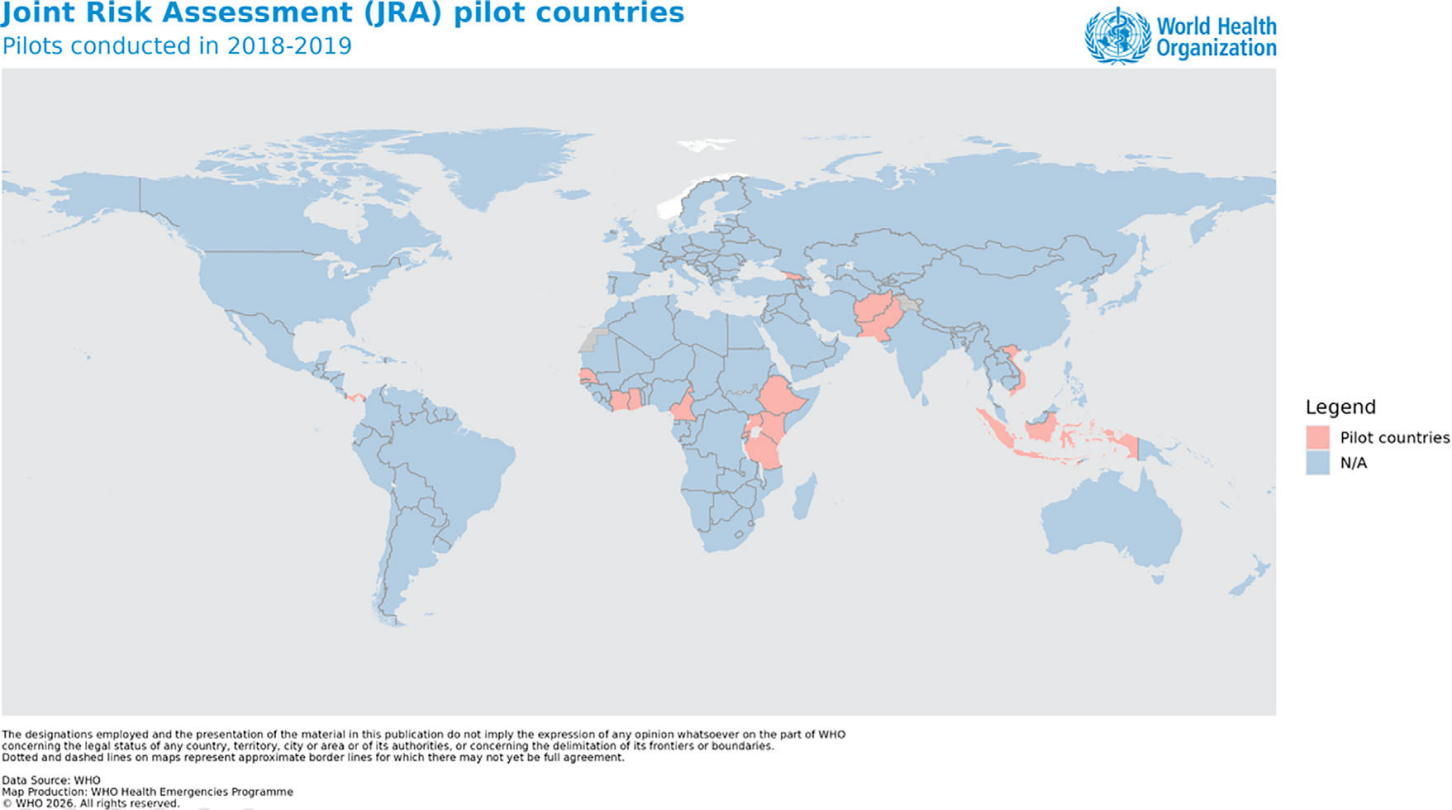
Joint Risk Assessment pilot countries.

**TABLE 1:** Objectives of the Joint Risk Assessment (JRA) pilot workshops.

Direct objectives	Indirect objectives
Build capacity through training a pool of national facilitators to operationalize the JRA tool at national and subnational levels Provide likelihood and impact estimates for risk assessment questions on priority hazards Assess the usefulness of the JRA and consider its sustained use Collect feedback to improve interim versions of the JRA	Enhance coordination and trust among relevant stakeholders Facilitate official and unofficial communication and information sharing among stakeholders Advocate for allocation of resources

**FIGURE 2:**
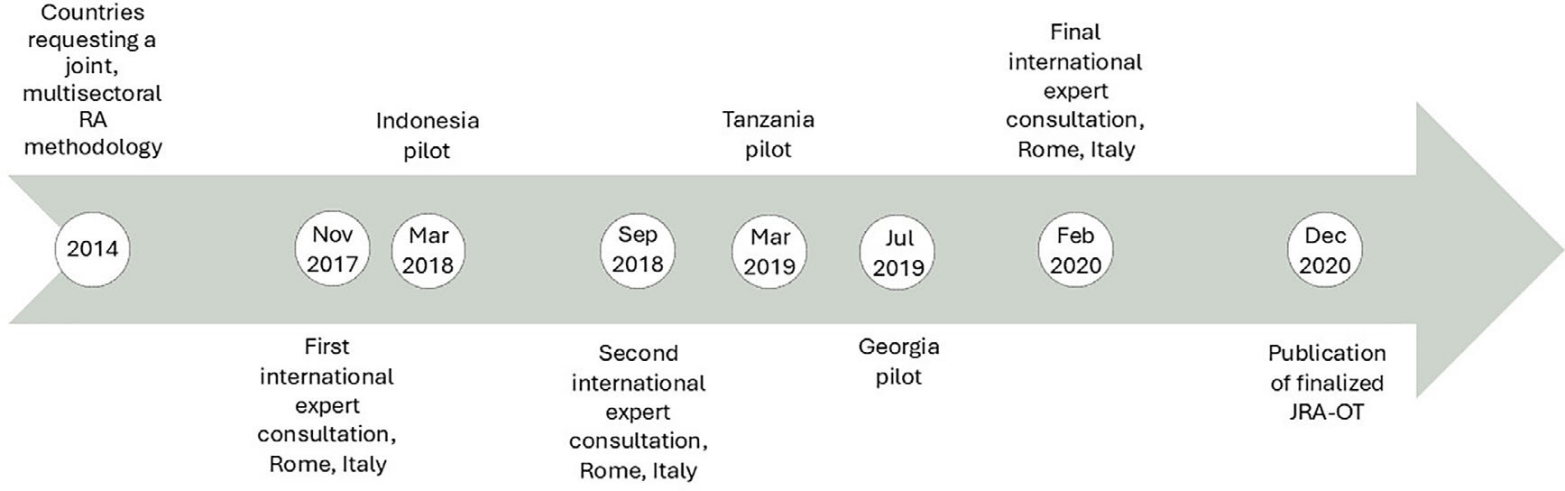
Development timeline of the Joint Risk Assessment operational tool.

### 2.2 Pilot Workshops

For each of the 15 pilot workshops, all relevant ministries, particularly those responsible for animal health and public health, were encouraged to engage through outreach by FAO and WHO country offices. Tripartite central, regional, and country office staff supported the workshops as external facilitators. All pilots, aside from the Indonesian pilot where the risk framing was conducted during the workshop, followed the six steps, below and in Figure 3, adapted from common RA processes used in public health, animal health, and/or environmental RA:

1. *Hazard identification*: Technical staff and decision‐makers in engaged ministries (hereafter, “ministries”) agreed on national priority endemic, epidemic, or emerging zoonotic hazards, up to 6 months prior to the workshop.
2. *Risk framing*: Once the hazards were agreed, ministerial decision‐makers characterized the scope, purpose, and objectives of the assessment on the basis of government strategy and policy to ensure the JRA process remained efficient and focused on critical government needs.
3. *Selection of participants and national staff*: After Steps 1 and 2, ministries identified participants based on the identified hazards and risk framing and selected staff to facilitate the planning and implementation of the pilot and future JRAs (hereafter, “national facilitators”). A JRA lead was agreed among ministries. High‐level ministerial participation was encouraged for workshop Days 0 and 3.
4. *Day 0*: National facilitators finalized the risk framing and were trained on the JRA process and tool by the external facilitators.
5. *Days 1–2*: The JRA was introduced and undertaken by participants, led by the national facilitators and supported by external facilitators. Small groups each focused on one identified hazard using information brought to the workshop and presented the outcome of each step in plenary. Substantial time was dedicated to plenary discussion and questions, covering both the technical assessments and the JRA process itself.
6. *Day 3*: Each group presented final results in plenary. Participants, including decision‐makers, considered existing national zoonotic disease and One Health infrastructure and options for governance of routine JRA leadership and implementation, and identified what criteria would trigger conducting or updating a JRA.

**FIGURE 3:**
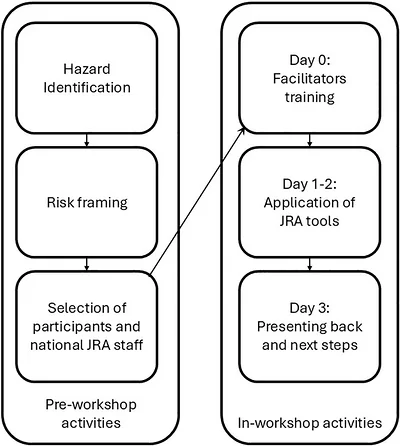
Schematic of the final Joint Risk Assessment process applied during national JRA pilots. Steps are fully described in WHO et al. [8].

### 2.3 Data Capture, Analysis, and Integration

From each pilot's planning period through to the end of each workshop, constraints, challenges, and proposed solutions gleaned from plenary and group workshop discussions, and verbal feedback from participants and national facilitators were captured in written notes taken by the external facilitators. Standardized written feedback on the process, tool, and workshop was requested anonymously from attendees using a questionnaire (Pilot Feedback Form; see Supporting Information). Completion of questionnaires was optional.

At the end of each workshop day, the external and national facilitators gathered to discuss how the day went and share the feedback received so far. Potential modifications to the JRA based on captured information and challenges were continually discussed among the pilot facilitators and the Tripartite focal points leading the development of the JRA (hereafter, JRA Secretariat). After each pilot workshop, the JRA Secretariat met virtually to revisit the feedback received and suggest corrective actions to the tool and process accordingly. On the basis of importance, urgency, and complexity, straightforward and uncontroversial corrections were made by the JRA Secretariat immediately and prior to the next workshop, whereas more complex issues were collected in a list of discussion points and brought forward to expert review and decision during the final expert workshop held in Rome (September 2018). As pilots were conducted, the JRA Secretariat noted that challenges fell into three categories:

- Challenges that were consistent across countries, such as misleading or unclear text, and definitely required changing to ensure comprehension.
- Challenges that varied across countries and could not be improved in a generic way and were best managed by stating in JRA materials that these aspects are country‐specific and would be managed during national JRAs.
- Challenges that fell outside the scope of the JRA process and would not be useful to address in the tool.

### 2.4 Country Pilot Selection for This Manuscript

The pilots in Indonesia, the United Republic of Tanzania, and Georgia (Table 2 and further described chronologically below) were purposively selected by the JRA Secretariat for further description in this manuscript, based on (1) each being the first country in its geographical region to conduct a JRA pilot and (2) the level of contribution the country's findings had in informing the final JRA content and processes.

**TABLE 2:** Summary of Joint Risk Assessment pilot workshops in Indonesia, Tanzania, and Georgia.

	Indonesia	Tanzania	Georgia
Dates	March 13–16, 2018	March 25–28, 2019	July 2–5, 2019
Location	Bogor	Dar es Salaam	Kakheti
Number of participants	64	24	25
Hazards assessed	Zoonotic influenza viruses, rabies virus, *Leptospira interrogans*	Zoonotic influenza viruses, rabies virus, *Bacillus anthracis*	Rabies virus, Crimean Congo hemorrhagic fever virus

## 3 Results

### 3.1 Indonesia

The Indonesian JRA pilot workshop was the first JRA pilot conducted and took place in Bogor on March 13–16, 2018. The 64 participants included representatives from central government ministries spanning human and animal health, forestry and environment, security and disasters, the Coordinating Ministry of Human Development and Culture, and health and livestock disease investigation staff from three provinces. Five hazards (zoonotic influenza viruses in three different contexts, rabies virus, and *Leptospira interrogans*) were assessed. Leveraging the strong multisectoral engagement and zoonotic disease experience of local partners facilitated the emergence of important lessons. Because Indonesia was the first pilot, these lessons greatly impacted the JRA process and tool and all subsequent pilots.

The Indonesia pilot generated key findings related to risk framing, pre‐workshop training, political will, and subnational involvement.

#### 3.1.1 Introducing a Risk‐Framing Step

Initially, the draft JRA process did not include a step where decision‐makers could provide strategic and policy‐related inputs to frame the assessment. As such, Indonesian participants and facilitators found they had to identify the assessment's scope and policy objectives as a first step of the group work. This was time‐consuming and only possible because ministerial decision‐makers were present and engaged to advise. The national facilitators suggested that ministerial decision‐makers could undertake this step prior to the workshop, inspiring the addition of the pre‐pilot risk‐framing step to the final JRA.

#### 3.1.2 Adding a 1‐Day Pre‐Workshop Training for National Facilitators

Initially, the “Day 0 national facilitators” training was also not part of the JRA process. Proactively, the Indonesian organizers initiated a full‐day pre‐pilot meeting of the JRA Secretariat and national technical staff to familiarize national facilitators with the JRA steps and to review the workshop objectives. These included staff from the provinces, and hereafter, the term “national facilitators” is inclusive of subnational staff. The entire team agreed that this pre‐pilot meeting was critical to the success of the workshop as it ensured that national facilitators (1) understood the JRA principles, step‐wise process, and tool; (2) practiced facilitation skills with support from the external facilitators prior to the workshop; and (3) could take a leading role in facilitation the next day. Skilled national facilitators would not only bring local context and perspectives but also create JRA sustainability, as external facilitators would no longer be needed for subsequent national and subnational JRAs. Subsequently, “Day 0” was incorporated for all future pilots, with national facilitators and JRA Secretariat attending Days 0–3 and all other participants attending Days 1–3. “Day 0” is not included in the tool as the JRA is meant to be done autonomously by countries, that is, once facilitators are familiar with the JRA tool and process.

#### 3.1.3 Importance of Political Will for JRA Success

The Government of Indonesia (GOI) was the first government to request a JRA workshop in 2017. The Ministry of Health (MoH), Ministry of Agriculture (MoA), and Coordinating Ministry of Human Development and Culture (Kemenko PMK) were strongly involved in planning and implementation. Director‐level representatives from these agencies were present during the entire workshop, contributed operationally to discussions, and were available to support the unanticipated risk‐framing activity. Not only did the Director General of MoH Prevention and Disease Control open the JRA, but he also requested a side meeting with the JRA Secretariat to show his direct support and appreciation of the JRA initiative and One Health approaches in general. The team agreed that this high‐level political engagement, as well as leveraging the strong national One Health culture, facilitated the later implementation of JRAs at national and subnational levels [10, 11]. The MoH proposed translating and adapting the tool further for the Indonesian context and embedding JRA within the national and provincial frameworks for zoonoses and other emerging infectious diseases based on the existing infrastructure and legal framework. This would be coordinated centrally by Kemenko PMK in collaboration with MoH, MoA, and Ministry of Environment and Forestry. Political will is now highlighted in the final JRA tool as a key element for enhancing the JRA success.

#### 3.1.4 Subnational Involvement for Field‐Context and to Ensure Relevance of Outputs

The proactive inclusion of participants from three provinces in the workshop permitted separate assessments of influenza viruses in three different contexts by participants living in those contexts. This allowed nuanced RA and creation of risk management options specific to their epidemiological and cultural context. It emphasized both that not all risks are national, and that local knowledge is critical to assessment of context‐specific risks. Participants were able to then implement subsequent provincial assessments autonomously.

#### 3.1.5 Subsequent JRA Implementation in Indonesia

To further support subnational JRAs, Indonesia initiated national JRA Training of Trainers (ToTs) activities in 2020 followed by ToTs in 10 provinces in 2021, which benefitted from the clear, stepwise guidance provided by the JRA‐OT. Subsequent JRAs applied the revised final tool and operational process and were conducted for SARS‐CoV‐2 virus (2021), *Mycobacterium bovis* (2022), avian influenza viruses (2022, 2023), rabies virus (2023), and Nipah virus (2026).

### 3.2 United Republic of Tanzania

The workshop in the United Republic of Tanzania, hereafter referred to as “Tanzania,” took place 1 year later, in Dar es Salaam on March 25–28, 2019. Tanzania was the first African country to conduct a JRA pilot workshop. Twenty‐four representatives of diverse government agencies, including disaster management, at central and subnational levels (e.g., Zanzibar), the One Health Coordination Desk (OHCD), and academic institutions participated during Days 1–3, whereas the JRA Secretariat, national facilitators, the One Health Coordinator, and the Minister of Health also attended Day 0 for the national facilitator training. Zoonotic influenza virus, *Bacillus anthracis*, and rabies virus were chosen as priority hazards. Both Tanzania's experience managing zoonotic diseases and the active OHCD for multisectoral coordination, which had experience organizing One Health trainings, facilitated success in pre‐pilot activities and workshop planning.

The Tanzania pilot validated key findings from the Indonesia pilot. As with Indonesia, Tanzanian participants suggested organizing nationally led ToT at the central and subnational levels. The importance of political will and high‐level input was affirmed, with ministerial participation on all 4 days of the workshop. Zanzibar was highlighted as a priority setting for implementing subnational JRAs, confirming their importance and value. Further findings included the use of virtual risk framing and using existing structures to sustain JRA implementation.

#### 3.2.1 Risk Framing Through Virtual Consultation

Tanzania was one of the first countries to conduct a risk framing and the first done virtually with support from the JRA Secretariat. Practical lessons were learned about risk‐framing implementation, for example, that each hazard should be framed separately, and that the risk‐framing template should contain only information strictly necessary to focus the RA. Importantly, Tanzania demonstrated that the risk framing was effectively done virtually, and benefits of the virtual risk‐framing meeting were noted by the JRA Secretariat.

#### 3.2.2 Utilizing the Existing Multisectoral Coordination Mechanism as JRA Steering Committee

Already in 2015, Tanzania had developed and implemented its Prime Ministerial level One Health Strategic Plan and had established the politically supported OHCD [12]. The existence of the OHCD facilitated the organization of the JRA, including convening appropriate participants and considering the 2017 Zoonotic Disease Prioritization Workshop [13] during hazard identification.

#### 3.2.3 Considerations for Subsequent JRA Implementation in Tanzania

During the pilot, the OHCD committed to functioning as Tanzania's JRA Steering Committee for continued use of the JRA and operationalizing JRA technical recommendations. The pilot workshop also revealed important country‐level challenges for collaboration that allowed for strategic national improvements. For example, despite ongoing disease‐specific efforts (e.g., for rabies), routine data sharing between sectors was previously recognized to be a gap. Creation of an information repository was recommended as an option on the basis of the results of the JRA and led to the establishment of a joint repository where information on priority zoonoses is accessible by all sectors.

### 3.3 Georgia

The workshop in Georgia took place in Kakheti on July 2–5, 2019. It was the first JRA pilot in Europe. The 25 participants represented the National Center for Disease Control and Public Health (NCDC), One Health Division and diverse government agencies. Approximately 50% of the participants came from seven subnational regions. Georgia had experience applying a One Health approach for zoonotic diseases and had already established a One Health Division within the NCDC, which supported JRA pilot planning and high‐level engagement. Georgia was the first country where the JRA Secretariat conducted a pre‐pilot mission to review the principles of the JRA and support pre‐pilot activities.

The Georgia pilot reinforced the iterative learning gained during the previous pilot workshops and highlighted the importance of ensuring that risk framings are done with strong high‐level input, given the impact they have on subsequent JRA steps.

#### 3.3.1 High‐Level Input Into Hazard Identification and Risk Framing Is Key

Prior to the pre‐pilot mission, MoH and MoA staff identified *B. anthracis* and *Brucella* spp. as hazards and conducted separate, sectoral risk framings for each hazard. During the pre‐pilot mission, the alignment of these hazards and risk framings with the national objectives for conducting the JRA was discussed. On Day 0 of the workshop, the Chief Veterinary Officer (CVO) and national facilitators again reviewed the hazards and risk framings. The national JRA team jointly proposed to change the hazards to rabies virus and Crimean‐Congo hemorrhagic fever (CCHF) virus, agreeing that the risks of anthrax and brucellosis, as endemic diseases, had been thoroughly assessed in the country and risk management plans were already in place. The importance of rabies to tourism and CCHF to trade were raised in the context of the economy and national policy. By the start of Day 1, the national facilitators had updated the risk framings for the new hazards. This experience emphasized the need for further clarification in the tool on the opportunities provided by conducting the JRA and resulted in the revision of the risk‐framing guidance in a step‐by‐step manner to ensure, for example, that risk framings are done jointly and with strong high‐level input.

#### 3.3.2 The Importance of Involving All Relevant Sectors

The pilot in Georgia highlighted the importance of ensuring that all relevant sectors are represented in the workshop. Participants recognized that not having the wildlife sector present during the discussion of rabies risks in rural areas limited the expertise and information available during the assessment. Given the last‐minute inclusion on Day 0 of rabies as a hazard to be assessed, the wildlife sector had not been identified as a key participant to be invited to the workshop. However, not only was the JRA process still possible without the wildlife sector present, but it was also useful in identifying the gap in sectoral participation and information available, making the JRA a tool for strengthening multisectoral collaboration overall.

#### 3.3.3 Political Engagement to Meet Policy Needs

The positive impact of high‐level engagement was also evidenced in Georgia by the participation of the CVO on Days 0–2 of the workshop and his enthusiastic engagement in both the technical and policy discussions. This allowed informed discussion to refocus the JRA to meet policy needs, reinforced the commitment of the participants to the assessments, and affirmed that their work was important and that the results would be used effectively.

#### 3.3.4 Subnational Engagement for Enhanced Collaboration Across Levels

During the workshop, the importance of assessing and managing rabies risks from wildlife to protect tourists visiting the mountainous regions of Georgia and reducing the impact on the tourism industry was highlighted, which was the focus of the revised risk framing.

#### 3.3.5 Considerations for Subsequent JRA Implementation in Georgia

National and subnational participants proposed to immediately use the JRA at the subnational level to better understand these specific risks and to develop local risk mitigation measures. This highlighted how including subnational participants in the central‐level JRA workshops ensures hazards are recognized and addressed across levels as well as strengthening multisectoral collaboration throughout the country.

These three pilot workshops demonstrated JRA's utility and provided critical information for improvements. A wide breadth of knowledge and experience was captured, from the key lessons highlighted above to detailed feedback on wording and presentation of the tool. In addition, after participating in a number of pilots, the JRA secretariat noted how effective the tool was at leading the participants and newly trained facilitators through the JRA process. Thus, we added the need for “clear, stepwise operational guidance” as a key finding, as it not only supports effective collaboration and supports ToTs, but is a concept transferable to co‐development of future operational tools.

Notable challenges not described above but addressed in the final tool included managing data gaps, clarifying risk characterization criteria within tables, clarifying JRA roles, writing suitable RA questions, and using standard templates for outputs. Other lessons (e.g., the value of pre‐pilot missions, effectively running “Day 0,” and English language interpretation), though critical for improving pilots, were not relevant to country‐led JRAs and thus not integrated into the tool.

## 4 Discussion

To address the lack of One Health operational tools to guide joint, multisectoral RA for zoonotic disease threats, the Tripartite co‐developed the JRA‐OT with inputs from 15 pilot countries and international experts. Between 2015 and 2020, feedback was incorporated iteratively into the JRA‐OT by a Tripartite Secretariat to generate the final version [8] published in 2020.

Pilots in Indonesia, the United Republic of Tanzania, and Georgia were the first to be conducted in their respective geographical regions and generated key lessons that were particularly important for informing the final tool and, thus, were selected for detailed description in this manuscript. There was much overlap across the findings from these countries, suggesting that they also represented countries generally. Key findings included the (1) value of policy‐level risk framing, (2) importance of political will and leveraging existing One Health culture from the three countries, (3) need for subnational engagement, (4) inclusion of all relevant sectors, and (5) necessity of providing clear, stepwise operational guidance and ToTs (Table 3).

**TABLE 3:** Summary of key lessons learned or confirmed across pilots and integrated into the final Joint Risk Assessment (JRA) tool and process.

Summary of key lessons learned or confirmed through JRA pilots	Lesson learned in		
	Indonesia	Tanzania	Georgia
Value of policy‐level risk framing (in person or online)	x	x	x
Importance of political will and leveraging existing One Health culture	x	x	x
Need for subnational engagement	x	x	x
Importance of including all relevant sectors			x
Necessity of providing clear, stepwise operational guidance and training of trainers	x	x	

### 4.1 Value of Policy‐Level Risk Framing

One of the most important findings across pilots was the need for a formal risk‐framing process led by decision‐makers ahead of the workshop. In Indonesia, participants realized that defining the scope, objectives, and policy priorities during the workshop itself was inefficient and only possible because senior officials were present. Tanzania further demonstrated that risk framing could be conducted effectively through virtual consultations and highlighted the importance of framing each hazard separately. In Georgia, better aligning the hazards and framing with national priorities related to tourism and trade on Day 0 reinforced the importance of strong policy input in hazard selection and framing prior to the workshop.

### 4.2 Importance of Political Will and Leveraging Existing One Health Culture

All three pilots showed that high‐level political commitment and existing multisectoral One Health coordination mechanisms were critical for successful implementation, as proposed by Yopa et al. [14]. Indonesia benefited from strong ministerial engagement and an established One Health governance structure, which later supported national and provincial JRA expansion. Tanzania's politically supported OHCD played a central role in organizing and sustaining JRA activities, whereas ministerial participation throughout the workshop reinforced national ownership. Similarly, Georgia's existing One Health Division within the NCDC facilitated planning, coordination, and policy relevance. Across pilots, political support increased participant engagement, improved alignment with national priorities, and facilitated translating JRA findings into action.

### 4.3 Need for Subnational Engagement

The pilots consistently demonstrated that involving subnational participants improved the relevance and practicality of assessments. During pilots, subnational participants were noted to be especially engaged and motivated to act. In Indonesia, representatives from three provinces assessed influenza risks in their own local contexts, enabling development of tailored risk management strategies and demonstrating that zoonotic risks are often context‐specific rather than purely national. Tanzania identified Zanzibar as a priority setting for future subnational JRAs, highlighting the value of localized implementation. In Georgia, regional participants emphasized differences in rabies epidemiology across areas and proposed immediate use of JRA methods at the subnational level. Overall, the pilots showed that subnational participation strengthens contextual understanding, improves local ownership, and supports implementation of locally relevant mitigation measures.

### 4.4 Inclusion of all Relevant Sectors

Another key lesson was the importance of ensuring representation from all relevant sectors in JRA workshops. Georgia particularly illustrated this challenge when the absence of the wildlife sector limited discussions on rabies risks involving wildlife transmission. This experience demonstrated that gaps in sectoral participation can reduce the completeness and quality of assessments. However, it also showed that the JRA process itself can help identify missing stakeholders and strengthen multisectoral collaboration over time. Across pilots, broad participation from human health, animal health, environment, disaster management, and other sectors was recognized as essential for comprehensive RA and effective One Health coordination for zoonotic diseases.

### 4.5 Necessity of Clear, Stepwise Operational Guidance, and ToTs

The pilots highlighted the need for practical, stepwise operational guidance and strong national facilitation capacity. Indonesia pioneered the Day 0 national facilitator training, which became a standard feature of subsequent JRAs. This training helped facilitators understand the methodology, practice facilitation skills, and eventually lead workshops independently, reducing reliance on external experts. Pilots in Tanzania and Georgia validated the value of this approach, with national facilitators increasingly taking ownership of workshop implementation. The Georgia pilot highlighted the need for clearer guidance on hazard selection and joint risk‐framing procedures that were then added into the tool. In both Indonesia and Tanzania, participants recommended national and subnational ToT programs to build sustainable in‐country expertise. Together, these experiences emphasized that successful JRA implementation depends not only on the tool itself, but also on clear, stepwise operational guidance, standardized processes, and national capacity building.

After review and discussion of these key lessons learned, the JRA Secretariat made the following revisions to the JRA tool and process: (1) introduction of a dedicated pre‐workshop risk‐framing Step 2 in the JRA process, led by decision‐makers, including a template for framing risks related to each hazard identified in Step 1; (2) highlighting the importance of political will and the opportunity to leverage existing national One Health structures to sustainably implement JRA; and (3) highlighting the importance of prospective inclusion of all relevant sectors, with participation of national and subnational levels, especially relevant in Step 3 where participants are being identified. The full‐day training for national facilitators on Day 0 was added to planning for all future workshops to foster ownership in country pilots, familiarize national facilitators, including subnational staff, with the JRA steps and some basic facilitation principles and review the workshop objectives.

All three countries saw the JRA as an opportunity to operationalize One Health strategies while producing practical, tangible outputs, specifically, consolidated hazard information, qualitative risk estimates, and options for risk‐based management and communication.

The evidence‐based JRA output served as advocacy to secure high‐level commitment and resources for risk mitigation. Participants from different sectors often still did not know each other, despite ongoing multisectoral activities and high levels of One Health awareness further emphasized the need to strengthen routine collaborative activities, such as JRA.

The JRA process can present challenges. Without strong One Health leadership or infrastructure, sectors may be reluctant or unable to commit time or resources or to share data, especially as assessments require updating as disease contexts change. Given staff turnover and required resources for ToT, trained national facilitators may be unavailable to initiate or update JRAs. However, guidance and training materials are available online [15] and via the standard support offered by the Tripartite agencies.

Despite these challenges, all three countries committed to undertaking JRAs for additional zoonotic diseases and at the subnational level. After the pilot, Balinese participants assessed risks from canine rabies in Bali, and risks from rabies and avian influenza were subsequently iteratively assessed in several other Indonesian provinces [10, 16, 17, 18] using the JRA. In 2021, Indonesia used the JRA to assess the risk of SARS‐CoV‐2 transmission from humans to dogs in DKI Jakarta. In Tanzania, based on a JRA recommendation, rabies awareness‐raising activities were coordinated jointly across sectors for World Rabies Day, 2019.

As of March 2026, 67 pilot and national JRA workshops have been completed in 41 countries across all six WHO regions [19]. The value and utility of the tool for conducting these assessments from the national perspective in diverse countries have been reported [20, 21, 22].

We recommend this approach to iterative co‐development and real‐world piloting of the JRA process and tool to be used as a model for co‐development of other multisectoral One Health operational processes and tools, to ensure an inclusive and consultative process consistent with the One Health approach.

## 5 Conclusion

Knowledge iteratively gained during pilots was critical to enhance the usefulness and effectiveness of the JRA process and tool, and ownership by different sectors. Key lessons can be grouped into the following categories:

1. The value of policy‐level risk framing
2. The importance of political will and leveraging existing One Health structures
3. The need for subnational engagement
4. The importance of all relevant sectors being engaged and present
5. The need for clear, stepwise guidance to facilitate efficient collaboration and support training of national trainers

The JRA pilots demonstrated that effective zoonotic disease RA requires strong integration of technical analysis with policy‐level decision‐making, particularly through early and structured risk framing. Sustained political commitment and established One Health coordination mechanisms were critical enablers of successful JRA implementation and long‐term national ownership. Inclusion of subnational actors and all relevant sectors significantly improved the contextual relevance, completeness, and operational value of RAs.

The pilots showed that JRAs can serve not only as technical tools for RA, but also as mechanisms for strengthening multisectoral collaboration, information sharing, and One Health governance. Standardized operational guidance, facilitator training, and nationally led ToTs were essential for building sustainable country capacity and reducing reliance on external support.

The JRA was co‐developed iteratively and inclusively across countries, sectors, and scales to meet objectives laid down by national governments and experts in RA and zoonotic diseases to assess risks, and thus models One Health best practice in both content and process and ensures ownership by the different sectors. The methodology can be used as a model for development of other multisectoral operational processes and tools. The JRA's inclusive, systematic, and evidence‐based RA process is now available to all countries, improving management of critical national zoonotic disease risks.

## Author Contributions

**Elizabeth Mumford** and **Sophie von Dobschuetz** originally conceived the JRA process and tool and led JRA development and piloting, with support from **Kachen Wongsathapornchai**. **Guillaume Belot**, **Dana Cole**, and **Dirk Pfeiffer** provided technical expertise. **Ryan Aguanno**, **Guillaume Belot**, **Elizabeth Mumford**, **Xavier Roche**, **Tieble Traore**, **Sophie von Dobschuetz**, and **Kachen Wongsathapornchai** led training workshops and pilots with national technical support from **Endang Wulandari**, **Vasily Esenamanov**, and **Ismaila Seck**. **Elizabeth Mumford**, **Sophie von Dobschuetz**, and **Kaylee Errecaborde** developed the manuscript, with support from **Ong‐orn Prasarnphanich**. **Elizabeth Mumford** and **Sophie von Dobschuetz** managed the manuscript publication process. All authors contributed to refinement of the JRA process and tool and/or implementing pilots, and all contributed to and reviewed versions of the manuscript, including the final version.

## Funding

The United States Agency for International Development (USAID), United States Centers for Disease Control and Prevention (CDC), United States Department of Agriculture (USDA), United States Defense Threat Reduction Agency (DTRA), and Korea International Cooperation Agency (KOICA) provided technical assistance, in‐kind funding, and/or material support.

## Disclosure

The findings, conclusions, and views expressed in this publication are those of the authors alone and do not necessarily represent those of the institutions with which the authors are affiliated. They should not be construed to represent any official WHO, FAO, USDA, or United States Government determination or policy.

## Ethics Statement

The activities described in this manuscript did not require submission to any ethical review board.

## Conflicts of Interest

The authors declare no conflicts of interest.

## Supporting information




**Supporting File:** puh270398‐sup‐0001‐SuppMat.pdf.

## Data Availability

Compliance with data sharing obligations is not applicable to this article as no datasets were generated or analyzed during the current study.
