# Supplementary material for: Iterative Co‐Development of the Tripartite Joint Risk Assessment Operational Process and Tool Through Incorporating Information From Pilots in Indonesia, the United Republic of Tanzania, and Georgia
Source: Public Health Chall. 2026 Oct 8;5(4):e70398. doi: 10.1002/puh2.70398 (PMC13650255; doi:10.1002/puh2.70398)
Supplement: Supplementary file 1 [file PUH2-5-e70398-s001.pdf]

DAY 1

Pilot Feedback Form

Tripartite Joint Risk Assessment Operational Tool pilot workshop

organizers/country offices please complete
place, date

THANK YOU

for completing this comprehensive Feedback Form. Your comments are critical to improve future workshops and improve the JRA Operational Tool before finalization.

1. ***My role in this workshop is: (circle one)***
   a. National participant
   b. National facilitator
   c. Regional Tripartite facilitator
   d. Other (please describe): ________________________________

Please note the three things you thought were the MOST useful about day 1 of the workshop?

2. a. --

3. b. --

4. c. --

Please note the three things you thought were the LEAST useful about day 1 of the workshop?

5. a. --

6. b. --

7. c. --

8. What else would have been useful to do today?

DAY 2

Pilot Feedback Form

Tripartite Joint Risk Assessment Operational Tool pilot workshop

organizers/country offices please complete
place, date

THANK YOU

for completing this comprehensive Feedback Form. Your comments are critical to improve future workshops and improve the JRA Operational Tool before finalization.

9. My role in this workshop is: (circle one)

e. National participant
f. National facilitator
g. Regional Tripartite facilitator
h. Other (please describe): _______________________________

Please note the three things you thought were the MOST useful about day 2 of the workshop?

10. a. --

11. b. --

12. c. --

Please note the three things you thought were the LEAST useful about day 2 of the workshop?

13. a. --

14. b. --

15. c. --

16. What else would have been useful to do today?

Questions about the JRA Operational Tool

17. *Please indicate the pathogen/hazard/event assessment you participated in during this workshop (e.g. XX, XX)(list each of the hazards assessed during the workshop)*

____

General Questions	Comment
18. *Was the JRA tool easy to follow and use? Please comment and assign a score based on*<br>*1 = No, not at all*<br>*5 = Yes, very*	
19. *What parts of the JRA tool were unclear?*	
20. *Are there specific changes that would make the JRA tool easier to follow?*	
21. *Were there specific areas / elements that did not work for the hazard you were assessing? If yes, what changes would you suggest?*	

JRA Tool specific sections and topics		
**Topic**	**Was this section or topic clear in the tool?**<br>Please assign a score based on<br>1 = No, not at all<br>5 = Yes, very	**What improvements could be made?**
22. *Terminology used and definitions*		
23. *Establish and convene a national JRA Steering Committee*		

24. Identify a JRA lead		
25. Establish and convene a JRA Technical Team		
26. Establish and convene a JRA Stakeholder group		
27. Risk framing		
28. Formulate and document risk assessment questions		
29. Identify and diagram the risk pathways		
30. Characterise the risk (assigning likelihood, impact, and uncertainty)		
31. Likelihood criteria table		
32. Impact criteria table		
33. Uncertainty criteria table		
34. Identify risk management options and communication messages		

35. *Document the assessment & Annex: Risk Assessment report template*		
36. *Operationalize risk assessment outcomes*		
37. *Annex: Information that may be required for the assessment*		
38. *Annex: Sources of information*		

39. *Do you have any other comments on the JRA Operational Tool?*

DAY 3

Pilot Feedback Form

Tripartite Joint Risk Assessment Operational Tool pilot workshop

organizers/country offices please complete
place, date

THANK YOU

for completing this comprehensive Feedback Form. Your comments are critical to improve future workshops and improve the JRA Operational Tool before finalization.

40. My role in this workshop is: (circle one)

i. National participant
j. National facilitator
k. Regional Tripartite facilitator
l. Other (please describe): _______________________________

Please note the three things you thought were the MOST useful about day 3 of the workshop?

41. a. ---

42. b. ---

43. c. ---

Please note the three things you thought were the LEAST useful about day 3 of the workshop?

44. a. ---

45. b. ---

46. c. ---

47. What additional topics would you have wanted to learn about today?

Please rate the statements below on a scale of 1-5 (If the statement is not relevant, check N/A):
1 = strongly disagree
2 = disagree
3 = neither agree nor disagree
4 = agree
5 = agree completely.

The direct objectives of the workshop were to:

- XX (organizers/country offices: please fill in agreed final objectives)

Indirect objectives of the workshop were to:

- get to know and trust colleagues from other sectors and disciplines
- make official and unofficial communication and information sharing among government agencies easier

**Objectives of the workshop**
1. *Which of the above objectives of the workshop were not achieved?* ____
48. *Which relevant ministries / sectors did not have appropriate representation?* ____

**Learning from the workshop**	**1**	**2**	**3**	**4**	**5**	**N/A**
49. *The trainers/facilitators knew the information and were able to answer questions*						
50. *The presentations were clear and helped me understand the topics*<br>51. *Please indicate the names of any trainers/facilitators or presentations that were particularly clear:* ____<br>52. *Please indicate the names of any trainers/facilitators or presentations that were particularly unclear:* ____						
53. *The group work helped me understand the topics*<br>54. *Please indicate group work that was particularly helpful:* ____<br>55. *Please indicate group work that was particularly unhelpful:* ____						
56. *I had enough time to raise questions and discuss in groups and the plenary sessions*<br>57. *Please indicate topics needing more time for discussion and questions* ____						

58. The presentations and facilitation were appropriate to the context of the country<br>*59. If NOT, why not?*<br>____						
60. The materials distributed were appropriate and useful<br>*61. Please indicate any that were not useful; and why:*<br>____						
62. As a result of my participation I feel confident to engage in risk assessments in my area of work						

**Post-workshop engagement**	1	2	3	4	5	N/A
63. As a result of my participation, I feel confident to contact staff from other offices in my sector to discuss shared issues (if you already did before the workshop, please tick here ( ) and rate any improvement in your confidence)<br>*64. If not, why not?*<br>____						
65. As a result of my participation, I now feel confident to contact staff from other offices in other sectors to discuss shared issues (if you already did before the workshop, please tick here ( ) and rate any improvement in your confidence)<br>*66. If not, why not?*<br>____						
67. As a result of my participation I feel confident to facilitate or advocate for better in-country communication and coordination between ministries involved in work on One Health and zoonoses<br>*68. If not, why not?*<br>____						

69. Any changes you would make to future workshops or other comments on the workshop itself?

